# Correlation analysis of whole genome sequencing of a pathogenic *Escherichia coli* strain of Inner Mongolian origin

**DOI:** 10.1038/s41598-024-64256-5

**Published:** 2024-07-05

**Authors:** Yan Jia, Kai Zhang, Jinshan Cao, Wei Mao

**Affiliations:** 1 Xuzhou Vocational College of Bioengineering, Jiangsu, 221006 Xuzhou China; 2 College of Veterinary Medicine, Inner Mongolia Agricultural University, Huhhot, 010018 Inner Mongolia China; 3 Key Laboratory of Animal Clinical Treatment Technology, Ministry of Agriculture, Huhhot, 010018 Inner Mongolia China

**Keywords:** *Escherichia coli*, Whole-genome sequencing, Antibiotic resistance genes, Virulence genes, Biological techniques, Zoology

## Abstract

Anal swabs of 1-month-old Holstein calves with diarrhea were collected from an intensive cattle farm, and a highly pathogenic *Escherichia coli* strain was obtained by isolation and purification. To study the virulence and resistance genes of pathogenic *E.* *coli* that cause diarrhea in calves, a strain of *E. coli* E12 isolated from calf diarrhea samples was used as experimental material in this experiment, and the virulence of the E12 strain were identified by the mouse infection test, and the whole genome map of the E12 strain were obtained by whole-genome sequencing and analyzed for genome characterization. The results showed that the lethality of strain E12 was 100%, the total length of E12-encoded genes was 4,294,530 bp, Cluster of Orthologous Groups of proteins (COG) annotated to 4,194 functional genes, and the virulence genes of sequenced strain E12 were compared with the virulence genes of sequenced strain E12 from the Virulence Factors of Pathogenic Bacteria (VFDB), which contained a total of 366 virulence genes in sequenced strain E12. The analysis of virulence genes of E12 revealed a total of 52 virulence genes in the iron transferrin system, 56 virulence genes in the secretory system, 41 virulence genes in bacterial toxins, and a total of 217 virulence genes in the Adhesin and Invasins group. The antibiotic resistance genes of sequenced strain E12 were identified through the Antibiotic Resistance Genes Database (ARDB) and Comprehensive Antibiotic Research Database, and it was found that its chromosome and plasmid included a total of 127 antibiotic resistance genes in four classes, and that E12 carried 71 genes related to the antibiotic efflux pumps, 36 genes related to antibiotic inactivation, and 14 antibiotic target alteration and reduced penetration into antibiotics, and 6 antibiotic resistance genes, and the resistance phenotypes were consistent with the genotypes. The pathogenic *E. coli* that causes diarrhea in calves on this ranch contains a large number of virulence and resistance genes. The results provide a theoretical basis for the prevention and treatment of diarrhea and other diseases caused by *E. coli* disease.

## Introduction

*Escherichia coli* (*E. coli*) is present in the intestinal tracts of humans and animals, and certain *E. coli* is one of the major pathogenic bacteria causing calf diarrhea, which leads to high morbidity, acute morbidity, high mortality, and extensive involvement in the disease, resulting in huge economic losses to the cattle industry^1^. Calf diarrhea (Calf diarrhea) is a harmful infectious disease, mainly caused by pathogenic *E. coli*^2^. It can lead to sepsis, enterotoxemia, and intestinal diarrhea in newborn calves, affecting the normal development of calves^3^. The rapid onset, seasonality, morbidity, and mortality of this disease require adequate attention in production^4^. However, there is a lack of effective treatment for the disease caused by this bacterium, and the clinical practice is mainly based on maternal antigens, antisera, and antibiotics, which are prone to drug resistance and drug residues^5^. Pathogenic *E. coli* invades host tissues and causes its pathogenesis by a variety of virulence factors coordinated with each other to play a role, the main virulence factors of *E. coli* include toxins, adhesins, outer membrane proteins, iron transport system and absorption system, and so on^6^. Enteropathogenic *E. coli* (EPEC) lesions in Attaching and effacing lesionsA/E Lesions (A/E) through the *E. coli* attaching and effacing factor (EaeA), which is responsible for encoding a protein located in the Locus In Attaching and effacing lesionsA/E Lesions (A/E), the protein is responsible for coding the Intimin^7^. *E. coli* can alter the structure of the target site of veterinary macrolides, consume energy to pump macrolides out of the body, and mutate N6 in A2058 of 23S rRNA to G/U to produce methylation or bimethylation, resulting in a decrease in the affinity of veterinary macrolides for the 50S subunit of the ribosome, and inactivation of the glycosylation catalyzed by the enzyme glycosyltransferase (GTase), which is a key component of macrolides' resistance mechanisms^8,9^.

*E. coli* excretes veterinary tetracycline antimicrobial drugs extracellularly via MF in EP, Ribosomal protection proteins (RPPs) binding to ribosomes can reverse distort the ribosomal structure, cause alterations in ribosomal conformation, and directly interfere with the interactions between the tetracycline D-ring and 16S rRNA base C1054 *E. coli* can also produce inactivating tetracycline enzymes and an unidentified mechanism to degrade tetracycline and render it inactive. The genes coding for them are tetA-E, respectively^10^. *E. coli* develops resistance through the alternative genes Sul1, Sul2, and Sul3 of Dihydropteroate synthase (DHPS) and the mutant gene folP. Sul1 is located in the conserved region of Tn21 of the integron (Integron, In) associated with the other ARGs, and sul2 is located in the ISCR2 element linked to the streptomycin resistance gene (aadA1)^11^. Mechanisms of polymyxin resistance in *E. coli* include the formation of BF and activation of Two-Component Systems (TCSs) on the LPS, leading to spatial conformational changes in the LPS overexpression of the extracellular membrane protein OprH, as well as a plasmid-mediated category (Mcrmobile colistin resistance, MCR)-/genes such as mcr-1, mcr-2 and the efflux pump AcrAB-TolC complex^12^.

Base mutations in *E. coli* through DNA helicases (gyrA, gyrB) and topoisomerase IV (two parC subunits and two parE subunits forming a heterotetramer of type A2B2) in the Quinolone Resistance Determining Regions (QRDR) result in heterologous tetramerization with the quinolone binding to be reduced, thus exhibiting no longer sensitivity to fluoroquinolones^13^. *E. coli* plasmid-mediated fluoroquinolone resistance is mediated by genes such as qnrA-D, qnrS, aac(6')-Ib-cr, oqxAB, and qepA, which act as "protectors" by interacting with quinolone targets^14–17^. *E. coli* produces β-lactamases that covalently bind to the carbonyl portion of the antibiotic, disrupting its ring structure and leading to degradation of the β-lactam antibiotic before it reaches the target^18^. *E.coli* develops resistance to aminoglycoside antibiotics through enzyme modification inactivation, which is accomplished primarily through aminoglycoside blunting enzymes present in the cytoplasm. For example, aminoglycoside phosphotran sferases (APHs), aminoglycoside acetyltransferases (AACs), and aminoglycoside nucleonucleotransferases (Aminoglycoside nucleon transferases (ANTs)^19^.

Pathogenic *E. coli* usually contain specific virulence-associated genes (VAGs), whose main purpose is to obtain nutrients for their proliferation from the organism, e.g., by moving in and out of the organism, VAGs are usually encoded on mobile genetic elements, such as extrachromosomal plasmids, phages, and different DNA fragments (gene islands), and they can also be transferred horizontally (propagated) between strains to generate new VAGs or change symbiotic (harmless) *E. coli* into pathogenic *E. coli*. Therefore, VAGs of pathogenic *E. coli* are the molecular basis of *E. coli* pathogenicity. Whole genome sequencing (WGS) can obtain nearly complete DNA information on pathogenic microorganisms^20^.

To study the causes of the high pathogenicity of pathogenic *E. coli* in this region, we used high-throughput sequencing technology to sequence the whole genome of one strain of highly pathogenic *E. coli* E12 and analyzed the genomic information, virulence genes, and antibiotic-resistance gene species of strain E12, to resolve the resistance mechanism and pathogenesis of calf diarrhea caused by pathogenic *E. coli* in this region.

## Results

### Results of drug sensitivity tests

By performing, on E12, the drug sensitivity tests in Table 2. The resistance rate of E12 to cefoxitin, penicillin, and ampicillin reached 100%. The resistance rate to ciprofloxacin, amoxicillin, ceftriaxone, and cefepime also reached more than 50%, and the sensitive drugs were meropenem, kanamycin, amikacin, polymyxin B, and ticarcillin-clavulanic acid, and the results are shown in Table 1.

**Table 1:** Drug sensitivity test results of pasture.

No	Abbreviation for drug resistance profile
E12	CIP-DOX-TET-FOX-KAN-CTR-CLM-CEL-AMP-LVX-T/S-ENR-PEN-FEP

### Results of pathogenicity tests in mice

**Figure 1:**
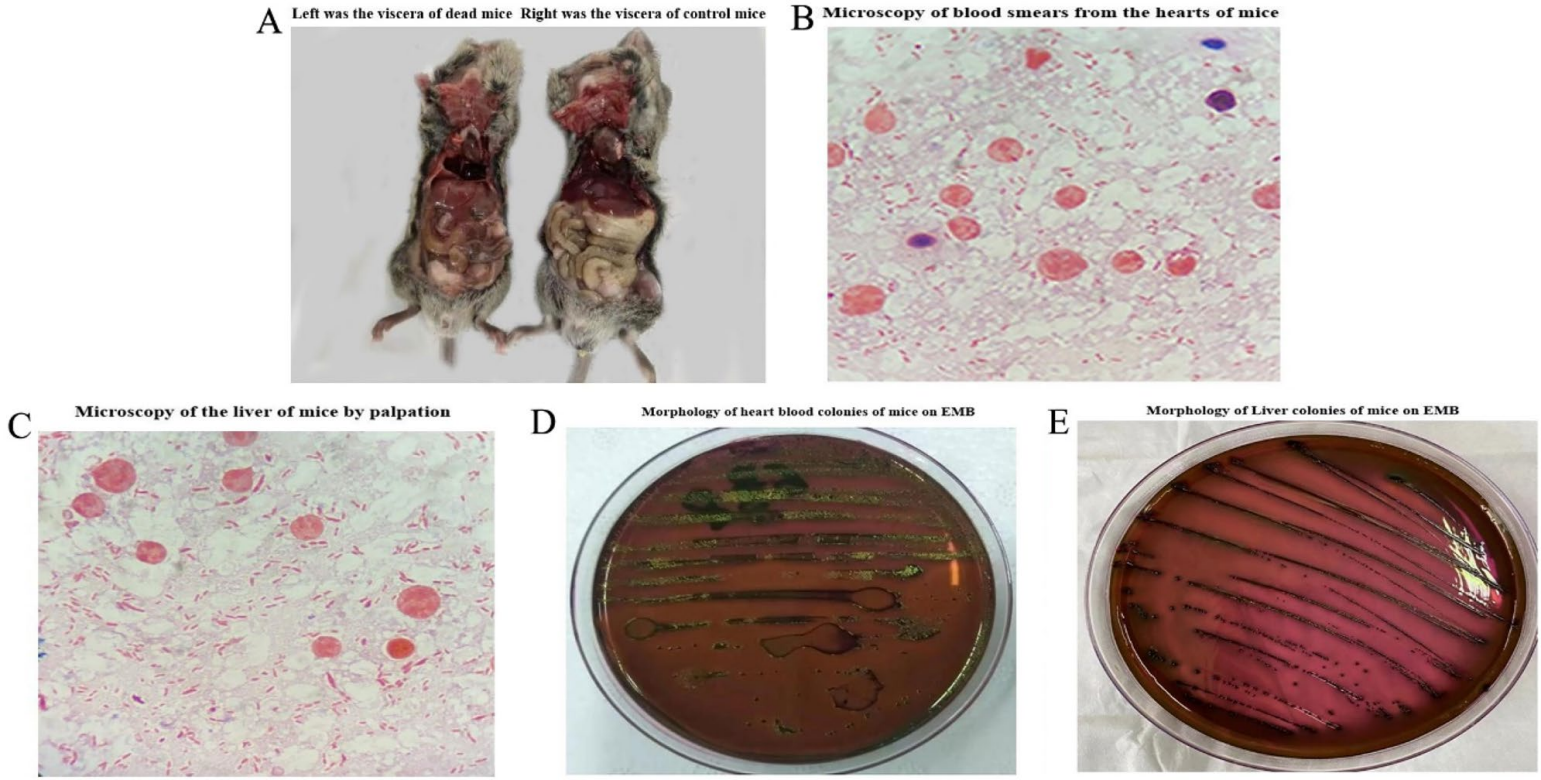
Results of mouse pathogenicity test of *E. coli* E12. (**A**) Left was the viscera of dead mice, right was the viscera of control mice; (**B**) Microscopy of blood smears from the hearts of mice; (**C**) Microscopy of the liver of mice by palpation; (**D**) Morphology of heart blood colonies of mice on EMB; (**E**) Morphology of Liver colonies of mice on EMB.

The pathogenicity of E12 was determined by the mouse pathogenicity test. Figure 1A (left) shows that after the death of mice caused by E12, the mice were dissected and showed subcutaneous hemorrhage in the abdominal cavity and different degrees of pathological changes in various organs. 1A (right) shows the viscera of control mice. Figure 1B shows a microscopic examination of blood smears from the hearts of mice killed by E12. Figure 1C shows a microscopic examination of liver smears from mice killed by E12. Figure 1B,C show the isolation of E12 from both the heart and liver of mice killed by E12. Figure 1D shows the colony morphology of blood from the heart of a mouse killed by E12 on an Erythromycin blue plate. Figure 1E shows the colony morphology of liver blood from mice killed by E12 on an Erythromycin plate.

### Results of genome assembly analysis

The E12 whole-genome sequencing results yielded 1367170reads, 16,409,118,810 bp of data, a Mean Read Length of 120023 bp, an N50 Read Length of 138100 bp, and a Mean Read Quality of 11.4. The E12 genome assembly results showed that it contained cyclized chromosome Chr1 and plasmid Plas1 with sequence sizes of 4763521 bp and 136382 bp, respectively, and GC contents of 50.88% and 49.98%, respectively (Table 2).

**Table 2:** Criteria for drug sensitivity test of *E. coli.*

Antibacterial drug	Drug content of paper tablets	Sensitivity Determination of Discount Points /(mm)			ATCC-25922
		S	I	R	
MEM	10 μg	≥ 23	20–22	≤ 19	28–35
AMP	10 μg	≥ 17	14–16	≤ 13	15–22
PEN	10 U	≥ 18	14–17	≤ 13	–
CEL	30 μg	–	–	–	15–21
FEP	30 μg	≥ 25	–	≤ 18	31–37
FOX	30 μg	≥ 18	15–17	≤ 14	23–29
CTR	30 μg	≥ 23	20–22	≤ 19	29–35
DOX	30 μg	≥ 14	11–13	≤ 10	18–24
TET	30 μg	≥ 15	12–14	≤ 11	18–25
LVX	5 μg	≥ 21	17–20	≤ 16	29–37
CIP	5 μg	≥ 26	22–25	≤ 21	29–37
ENR	5 μg	≥ 18	15–17	≤ 14	–
AMK	30 μg	≥ 17	15–16	≤ 14	19–26
ANA	30 μg	≥ 18	14–17	≤ 13	17–25
GEN	10 μg	≥ 15	13–14	≤ 12	19–26
T/S	23.75 μg	≥ 16	11–15	≤ 10	–
ERM	15 μg	–	–	–	–
CLM	30 μg	≥ 18	13–17	≤ 12	21–27
FFC	30 μg	≥ 18	13–17	≤ 12	–
POL	300 U	≥ 12	9–11	≤ 8	13–19

Sensitivity (S); Intermerdiary (I); Resistance (R).

### Results of genomic component analysis

According to Table 3, the E12 genome was predicted to have 4697 coding genes with a total gene length of 4,899,903 bp. 107 long terminal repeats (LTR), 30 DNA transposons, 46 long scattered in repeat (LINE), 25 short scattered in repeat (SINE), and 2 rolling circles (RC) were predicted for E12. The E12 genome contains 22 rRNAs and 88 tRNAs and 0 sRNAs in ncRNAs. 17 putative GIs, 18 Prophages, and 3 CRISPRs are predicted for E12.

The E12 genome assembly results showed that contained the circularized chromosome Chr1 and plasmid Plas1, in which the sequence sizes were 4,763,521 bp and 136,382 bp, with GC contents of 50.88% and 49.98%, respectively.

### Results of gene function analysis

**Figure 2:**
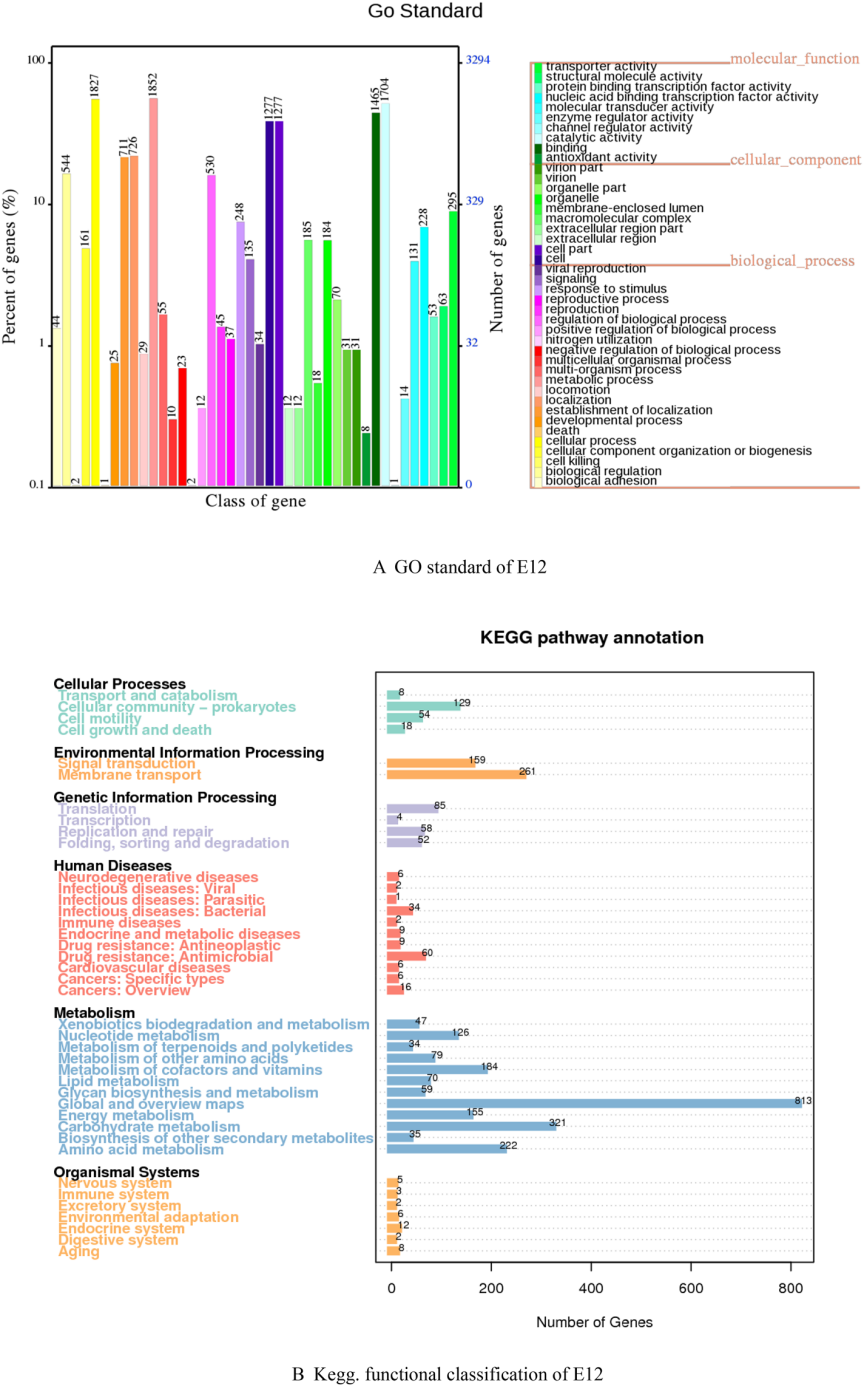

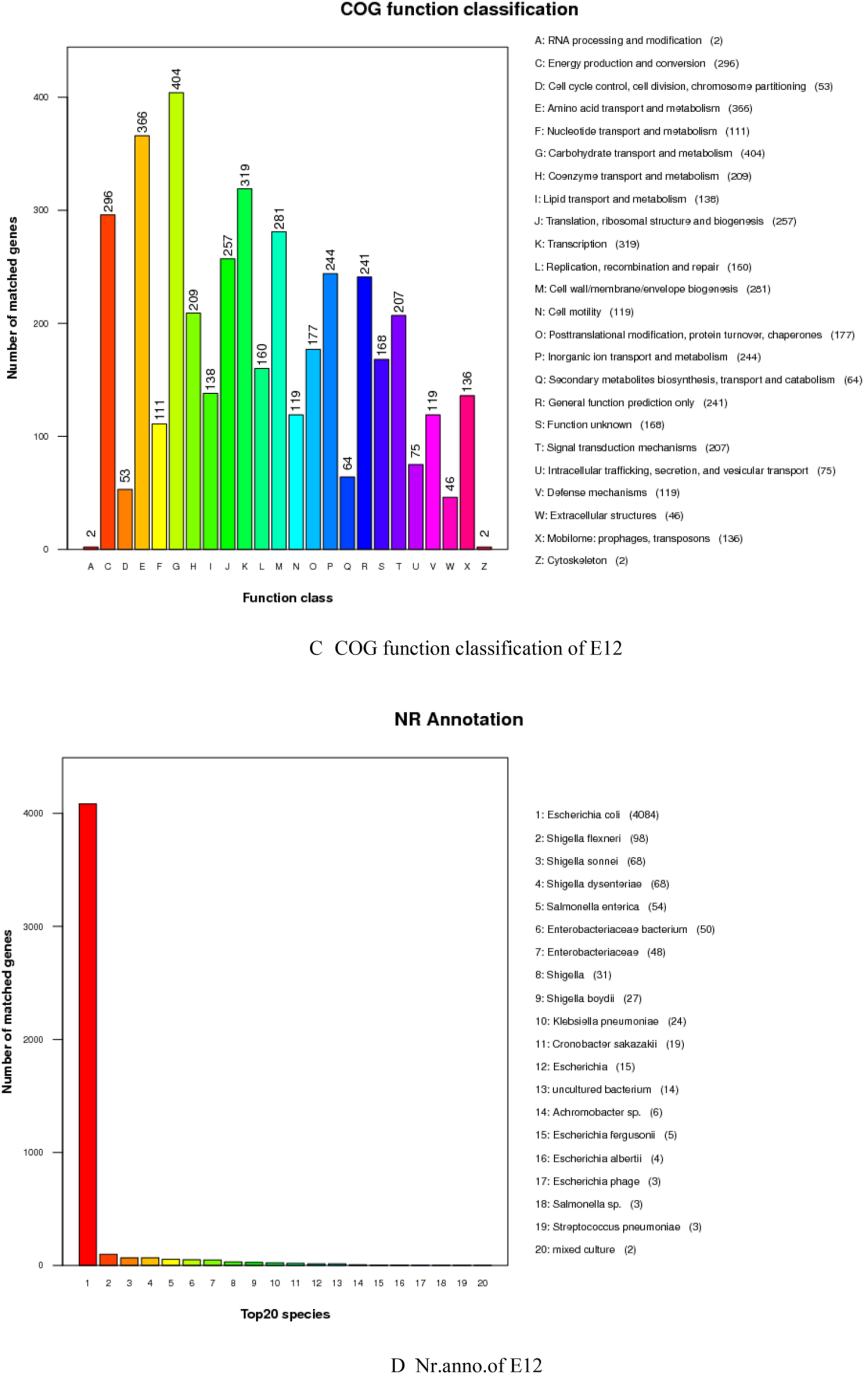

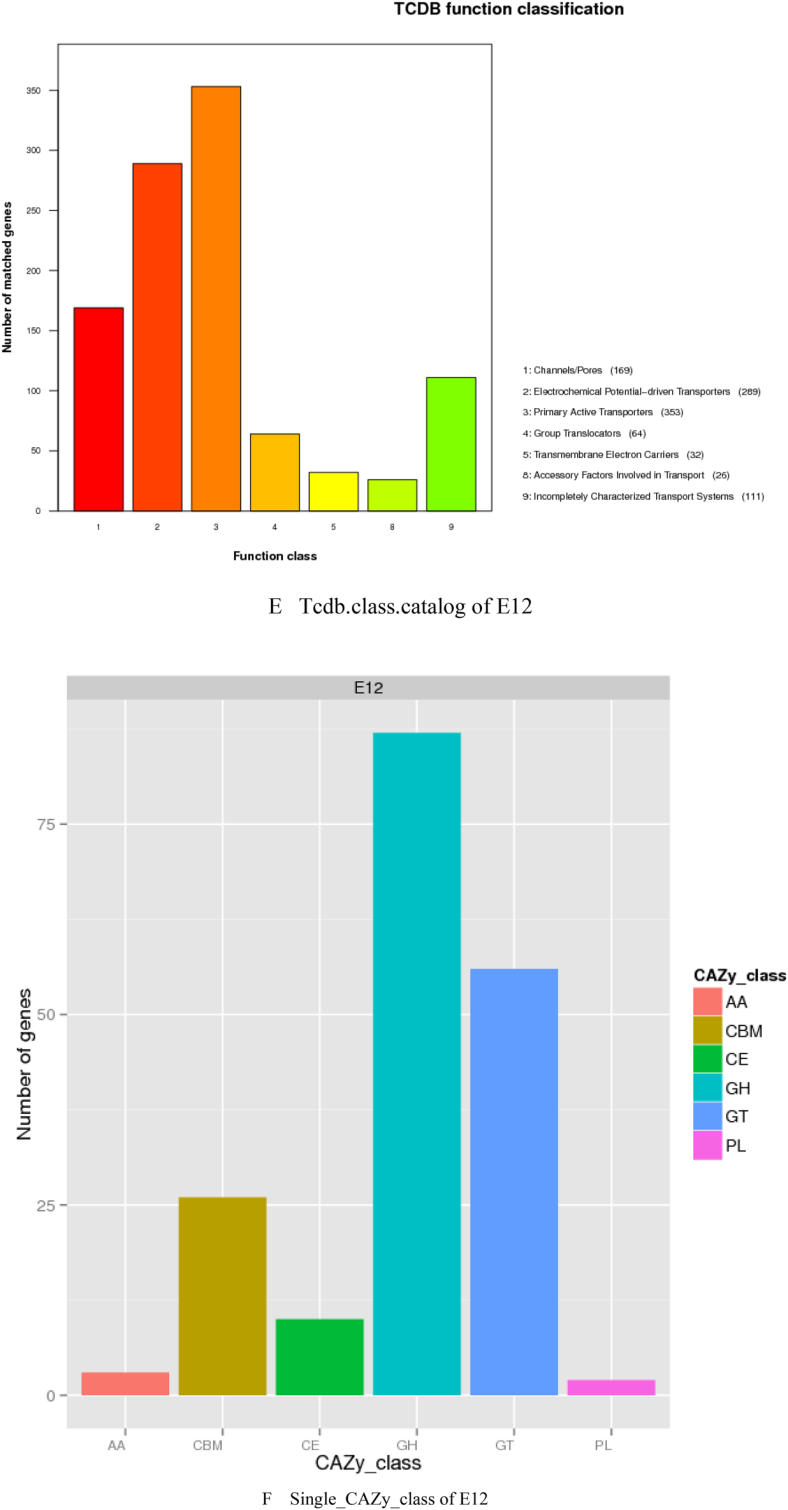

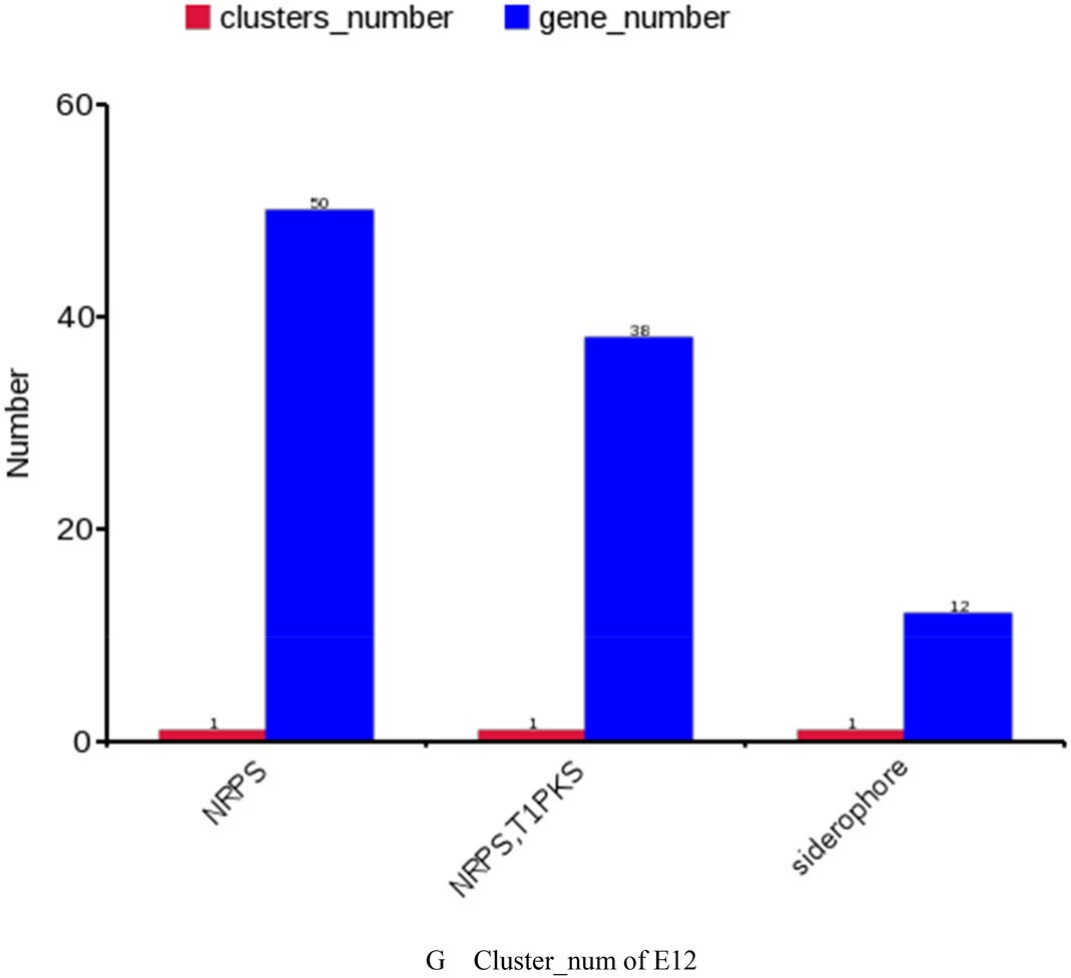
Gene function analysis of E12. (**A**) GO gene functional classification of E12; (**B**) KEGG metabolic pathway classification of E12; (**C**) COG functional classification of E12; (**D**) NR database species annotation of E12; (**E**) TCDB functional classification of E12; (**F**) Classification diagram of the KEGG metabolic pathway of E12; (**G**) Classification diagram of the KEGG metabolic pathway of E12.

The genomic score of E12 was analyzed through the database in Table 4, as shown in Fig. 2. As shown in Fig. 2A, a total of 14,112 genes were annotated to 43 functions by the GO database for E12. Among them, 3097 genes were annotated in Cellular Components, 7053 genes were annotated in Biological Processes, and 3962 genes were annotated in Molecular Function. As shown in Fig. 2B, 3160 genes were annotated by the KEGG database for E12, which were enriched in 40 metabolic pathways in six aspects, including Cellular Processes, Metabolism, and Organismal Systems. Figure 2C shows that 4194 genes of E12 were classified into 24 classes by COG as shown by the annotation results of the COG database. Figure 2D shows that E12 belongs to E. coli and contains 4084 genes as predicted by the NR database. Figure 2E shows that 169 Channols/Pores, 289 Electrochemical Potential-driven Transporters, 353 Primary Active Transporters, 64 Group Translocators, 32 Transmembrane Electron Carriers, 26 Accessory Factors Involved in Transport, 111 Incompletely Characterized Transport E12 has 6953 genes distributed in 343 protein families. A total of 4162 genes were obtained by annotation based on homologous genes. As shown in Fig. 2F, three Auxiliary Activities, 26 Carbohydrate related molecules, 10 Carbohydrate Esterases, 87 Glycoside Hydrolases, 56 E12 contains 392 signal peptide structural proteins, 1105 transmembrane structural proteins, and 32 secretory proteins. E12 was predicted to contain 6 T2SS secretion system effector proteins and 160 T3SS secretion system effector proteins by the secretion system TNSS (type N secretion systems). Figure 2G shows that E12 was predicted to have three clusters of secondary metabolic genes, including 12 siderophores, 50 Non-ribosomal peptide synthase (NRPS), and 38 Type I polyketide synthase (TIPKS).

### Annotation of virulence genes

**Figure 3:**
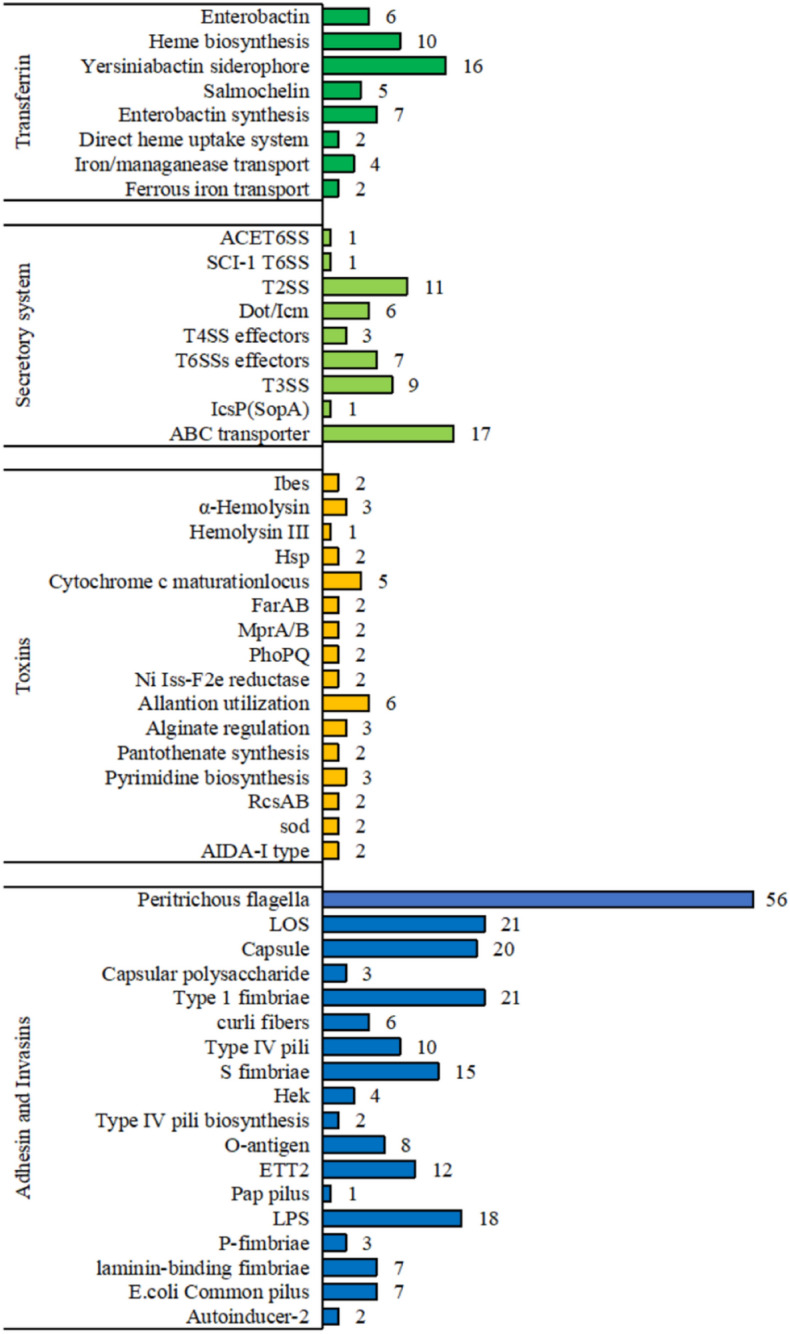
Virulence genes classification of E12.

The E12 virulence and resistance genes were functionally annotated according to the database provided in Table 3. Figure 3 shows that E12 obtained a total of 366 major virulence genes and related virulence genes by VFDB database matching. Among them, 52 virulence genes were found in Transferrin, 56 virulence genes were found in the Secretory system, 41 virulence genes were found in Toxins, and 217 genes were found in Adhesin and Invasins.

### Annotation of antibiotic resistance genes

**Figure 4:**
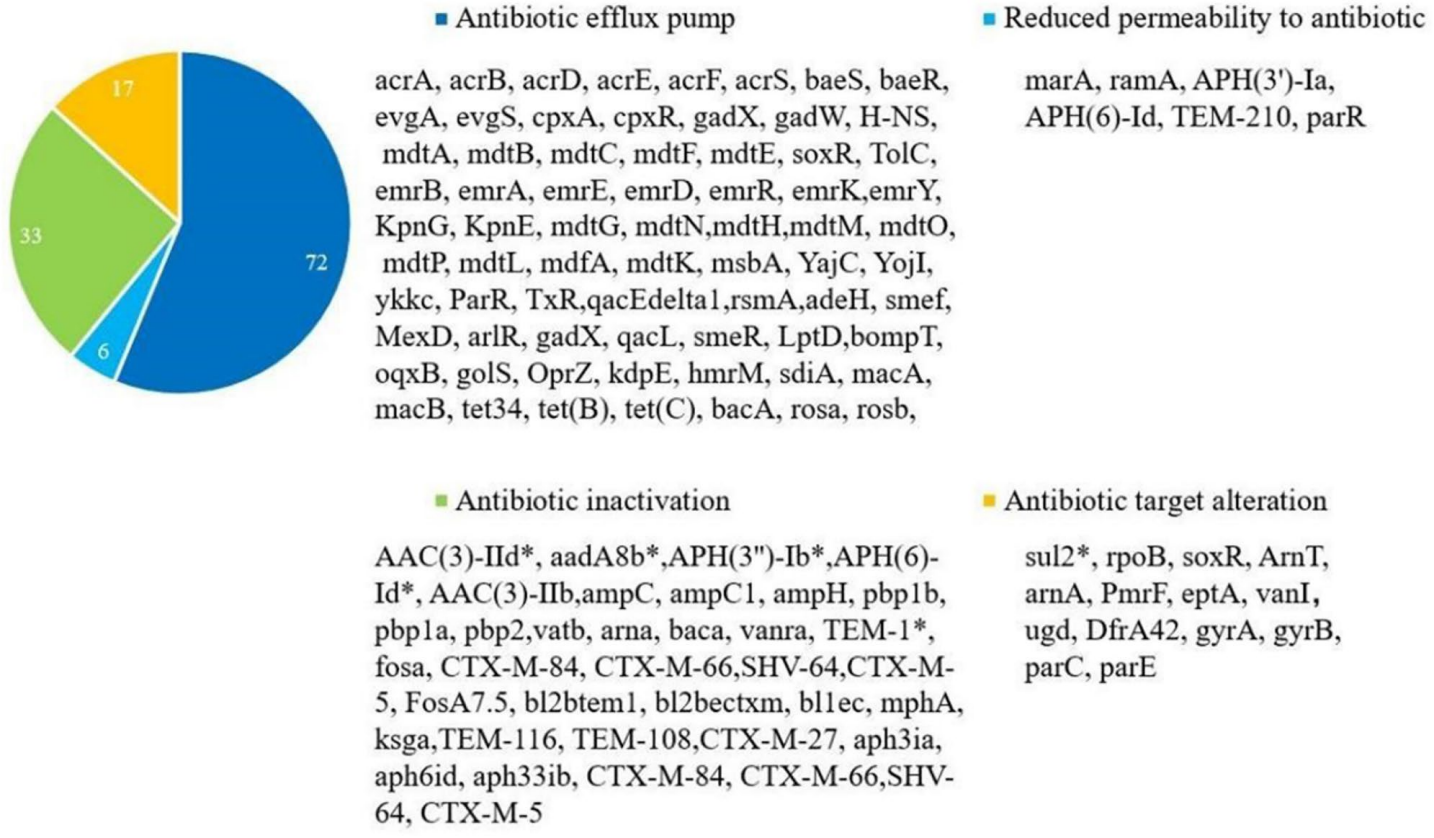
Antimicrobial resistance genes classification of E12. *Note*: *represents plasmid resistance genes.

As shown in Fig. 4, according to the comparison results of the ARDB and CARD database for E12, E12 was found to carry 71 genes for the antibiotic efflux pump system, 36 antibiotic inactivation-related resistance genes, 14 and 6 resistance genes for antibiotic target alteration, and reduced permeability to antibiotics, respectively, with a total of 127 resistance genes in 4 major categories. Among the plasmid-carried resistance genes were *TEM-1*, *AAC(3)-IIb*, *aadA8b*, *APH(3'')-Ib*, *APH(6)-Id*, and *Sul2*.

## Discussion

### Analysis of genomic features

The genome sequence of E12 as a drug-resistant strain can provide a control for the study of other susceptible *E. coli* strains. Compared with the results of previous papers, there is no significant change in the genome assembly of E12. The functional classification of E12 genes includes virulence genes and drug-resistant genes related to cellular biological processes, which are mainly involved in adhesion, metabolism, and stress response. The metabolic pathway classification of E12 focuses on metabolism, genetic information processing, signaling, and cellular processes. The metabolic pathways of E12 focus on metabolism, genetic information processing, signal transduction, and cellular processes.

### Relationship between drug resistance and genotype

In this study, we applied the CARD database to predict a total of 127 drug resistance genes on the E12 genome and plasmids, which are involved in all common current drug-resistance mechanisms^21^. The plasmid carrying resistance genes *AAC(3)-IId*, *aadA8b*, *TEM-1*, *sul2*, *APH(3")-Ib*, *APH(6)-Id* and chromosome carrying *ampC*, *ampH*, *pbp2*, *gyrA* in E12 corresponded to penicillin, aminoglycoside, anti-folate, tetracycline, macrolide, and cephalosporin antibiotics, respectively consistent with drug-resistance. No *blaOXA* and *aac(6)-Ib-cr* type β-lactamase genes were detected from E12, and only one related gene was detected for *blaSHV*, which may reaffirm the current dominance of *CTX-M* and *TEM* type ESBLs in *E. coli*^22^. This discrepancy in phenotypic and genotyping results may be due to the lack of gene expression in genetically predicted resistant but phenotypically sensitive strains to infer resistance^23^.

### Multiple drug resistance mechanisms

The E12 chromosome and plasmid in this study carry a total of 127 resistance genes, including *ampC*, which inactivates antibiotics, and *pbp2*, which alters the target of drug action. H-NS, *evg* genes combine both resistance nodal differentiation family (RND) and major susceptible subsuperfamily (MFS) resistance mechanisms^24^. The exclusion pump T'olC gene of the outer membrane combines three resistance mechanisms, RND, MFS, and the ATP-binding cassette family superfamily (ABC)^25^. E12 detected *marA*, the core gene of multiple antibiotic resistance (Mar), which has a mechanism to reduce antibiotic permeability in addition to the antibiotic efflux mechanism^26^. E12 contains *mdtH*, *emrB*, *emrA*, and other multi-drug resistance efflux pump system genes. *AcrR* genes combine antibiotic efflux and altered target of action mechanisms^27^. The *SoxR* gene has an altered antibiotic targeting mechanism in addition to an antibiotic efflux mechanism^28^。AcrAB-TolC belongs to the proton-dependent efflux pump (EP) system, which consists mainly of an intracellular membrane pump (AcrB) connected to an outer membrane exclusion pump (TolC) via a transmembrane fusion protein (AcrA) and is capable of mediating high levels of resistance to a variety of antimicrobial drugs^29^. E12 Many resistance genes are located on mobile DNA elements, making it possible for resistance genes to expand the host range of resistance transmission and for increased resistance genes to lead to the development of multiple drug resistance. The diverse antibiotic efflux system suggests that E12 has been in a high antibiotic concentration environment, and the multidrug resistance characteristics have been formed under huge selection pressure. High-throughput sequencing technology not only plays a crucial role in the discovery of new resistance genes, the mechanism of transmission of resistance genes, drug-resistant bacteria, and the evolutionary analysis of resistance genes, but also predicts removable elements with horizontal transfer functions, including genomic islands, CRISPR-Cas systems, and prophages. In this study, the whole gene sequencing of E12 not only identified a large number of virulence genes and resistance genes, and a variety of transposon elements and type I integrons were found in both the fec locus, HPI, and upstream and downstream of the resistance genes in the plasmid.

### Pathogenic mechanism

The pathogenicity of *E. coli* is produced by multiple virulence factors working in concert with each other. In this study, the virulence genes of E12 were analyzed using the VFanaIyzer online analysis tool provided by the VFDB database, and E12 carried a total of 366 virulence genes. Heme is the most abundant resource of iron ions in the host body, mainly by the *chuA* gene to take up iron from extracellular or host heme and iron in hemoglobin. E12 was detected to contain heme synthesis-related genes *hemA/B/C/D/E/H/G/L/N/X*, yersinia cepacia synthesis-related genes *fyuA*, *ybtA/E/S/T/U/Q*, *irp2*, *iutA*, and *iucA/B/C/D*. Fe/Mn transport protein is a toxicity factor of the iron uptake transport system, which is encoded by four genes, *sitA*, *sitB*, *sitC*, and *sitD*. *sitB* is an ATP-binding protein in the system, so it is equivalent to an ATP ion pump, which can provide the energy needed for the bacterial transport process. The outer membrane transport system can also load divalent iron into the cell. The outer membrane transport system can also load divalent iron into the cell, which is involved in nutrient uptake, secretion of bacterial toxins, and pumping antibiotics out of the cell to make bacteria resistant to drugs. The iron/manganese transport protein was present in the virulent strain E12 in this study. The Hemolysin gene (*hlyA*, *hlyB*, *hlyC*, *hlyD*, *hlyE*/*clyA*) is a member of the family of formation proteins that lyse mammalian erythrocytes and kills them by forming pores in the target cell membrane. A total of 41 virulence genes of bacterial toxins (Toxins) were detected in strain E12. Among the hemolysins, Alpha-hemolysin contained *hlyA*/*E*/*F* genes and Hemolysin III contained *B5650799* genes^30^. The genes involved in bacterial toxins are mainly Allantion utilization encoding genes, and the virulence factors of the iron transporter system are mainly Yersin synthetic virulence factors, these genes determine the virulence of E12. The types of virulence genes carried by E12 and the pathogenic mechanism are speculated that E12 may have certain public health risks. In this study, a total of four types of secretion systems were identified as ABC transporter protein of type I secretion system, type II, type III, and type VI, of which type III secretion system is widely present in Gram-negative pathogenic bacteria. Pathogenic bacteria inject specific proteins directly into host cells with the help of a secretory system, which can lead to bacterial colonization and infection^30^. Type III secretion system effectors (Non-LEE encoded effector, *Nle*) *NleH* and *NleD* have anti-apoptotic activity, *NleH* can promote cell survival and inhibit enterocyte loss, thus maintaining *E. coli* stoichiometry. During the infection phase, *E. coli* surface molecules induce exogenous apoptosis, and type III secretion system effectors such as *EspF*, and Map induce endogenous apoptotic pathways. *EspF* induces mitochondrial lysis, disrupts cellular tight junctions, and promotes the degradation of anti-apoptotic proteins. E12 has been detected to contain a type III secretion system (T3SS), including *espR1*/*L4*/*L1*/*X5*/*X4*, *spa40*, *hopAN1*, *and ipaH*/*H2.5* genes. The type VI secretion system (T6SS) is a novel secretion system ubiquitous in Gram-negative bacteria and was first identified in *Vibrio cholerae*. The T6SS is involved in bacterial biofilm formation and mediates adhesion and virulence between bacteria and their hosts. In this study, we detected 15 genes in ACE T6SS and *EC559893318* gene in SCI-I T6SS of T6SS^31^. The *E. coli* type III secretion system 2 (ETT2) virulence island of E12 was lost. This is consistent with the results that have been reported, where genetic analysis showed that almost most of the enteropathogenic *E. coli* ETT2 integrity was lost to varying degrees^32^. E12 was detected in adhesin and invasin-like virulence genes containing autoinducer-2 including *IuxS* and *eaeA* genes. The tight adhesin eae enables bacteria to adhere tightly to intestinal epithelial cells. eae binds to the tight adhesin translocation receptor protein Tir causing further signaling, a sudden increase in intracellular calcium ion concentration, and an increase in intestinal fluid secretion, resulting in the characteristic adhesion and effacing lesion (A/E). The virulence factors involved in adhesion and invasion of E12 were mainly type I hairs, periphytic flagella, lipooligosaccharides, and many other genes encoding them. *fimA*/*B*/*C*/*D*/*E*/*F*/*G*/*H*/*I*/*S*/*Z* 11 genes encoding type I hairs were detected in E12, and these flagellar genes play a role in coordinating the assembly of hairs and the pathogenic process^33^. The results showed that the characterization of the E12 virulence factor was close to that of published virulence factors for pathogenic *E. coli*.

The results of this experimental study indicate that the complex evolutionary history of E12 is conducive to a more comprehensive understanding of the characteristics of the genome of local strains, which can provide a reference basis for the treatment of infection and long-term prevention and control of local pathogenic *E. coli*.

## Materials and methods

### Sequencing strain

Anal swabs were collected from 1-month-old Holstein calves with diarrhea at an intensive cattle farm. Thirty-five samples were scribed and inoculated in ordinary broth medium to complete proliferation, and the morphology of the samples was similar to that of *E. coli* was streaked and inoculated on Eosin-methylene blue medium (EMB) plates and MacConkey Agar (MAC) plates. Then the preliminary determination of *E. coli* were subjected to Gram stain microscopy. After isolation and purification of the anal swabs, a strain of *E. coli* was obtained, which was named E12.

### Drug sensitivity test

Drug susceptibility testing was performed on sequenced strain E12 by referring to the Kirby-Bauer disc agar diffusion method (K-B) recommended by the Clinical and Laboratory Standards Institute (CLSI)^34^. The concentration of *E. coli* bacterial liquid to be tested was adjusted to 1.5 × 10^8^ CFU/mL by turbidimeter, and three replicate groups were made for each strain. ATCC 25,922 was used as the *E. coli* drug sensitivity quality control control group, and the criteria for determining the drug sensitivity test are shown in Table 2.

### Mouse pathogenicity test

We randomly divided the mice into three test groups (replicate groups) and one control group of four mice each. In this experiment, we used 6-week-old, physically healthy, clean-grade Kunming mice, half male and half female (16 mice in total), weighing approximately 20–22 g. The mice in the test groups were given 0.4 mL of E12 at a concentration of 1.5 × 10^8^ CFU/mL, while an equal amount of saline was given to the mice in the control group. Mice are infected by intraperitoneal injection of pathogenic *E. coli*. We observed the mice continuously for 3 d, recorded the time points of onset and death, and dissected the mice immediately after death to complete clinical observation and microscopic examination.

Mice infected with E12 showed a series of pathological changes a few hours later, such as diarrhea, unresponsiveness, loss of appetite, depression, etc. Smear staining and microscopic examination of tissue cultures of the liver and heart of infected mice showed blunt-rounded, gram-negative, non-budding bacilli at both ends.

Infected mice were found to have a large amount of mucus in the thoracic and abdominal cavities after an autopsy; yellowish-white dilute fecal contamination around the anus, and the intestinal lumen was filled with fishy yellow dilute contents; the liver and spleen were enlarged and bruised, and there were grayish-white necrotic foci on the surface of the liver and in the cut surfaces; splenomegaly was obvious, and there were large amounts of bruising and inflammatory necrotic foci of different sizes; the lungs were bruised, and there was dilatation of the capillaries in the alveoli with congestion and the appearance of cellulosic-like ooze The lungs showed bruising, dilated and congested capillaries in the alveoli, fibrinoid exudation, and intravascular thrombus; the intestinal epithelial cells were necrotic, detached and infiltrated with inflammatory cells.

### Translated with DeepL.com (free version)

#### DNA extraction

The genomic DNA of E12 was extracted by SDS, and then the purity and integrity of the DNA were detected by agarose gel electrophoresis (80 V, 30 min) and quantified by Qubit. The DNA was sent to Novogene for whole-genome sequencing.

### Whole genome sequencing and genome assembly

After sequencing E12 through the Nanopore PromethION platform, the genome was assembled using Unicycler software (https://github.com/rrwick/Unicycler) using second-plus-third generation high-quality reads to screen chromosome and plasmid sequences and assemble the chromosome sequences into a loop genome^35^.

1. Pacbio platform library construction and library inspection

The 10 K SMRT Bell library was constructed using the SMRT bell TM Template kit (version 1.0), and the electrophoretically detected DNA samples were interrupted by Covaris g-TUBE to form the target fragments of the required size for the library construction, and then DNA damage repair and end repair were performed, and DNA adhesion enzyme was used to connect the hairpin junctions at both ends of the DNA fragments.

(2) Illumina platform library construction and library inspection

The DNA samples that passed the electrophoresis test were randomly interrupted into fragments of about 350 bp by Covaris ultrasonic crusher. The DNA fragments were processed using the NEBNext®Ultra™ DNA Library Prep Kit for Illumina (NEB, USA).

After the library inspection was qualified, the different libraries were subjected to PacBio Sequel and Illumina NovaSeq PE150 sequencing according to the effective concentration and target downstream data volume.

### Genome component analysis

See Table 3.

**Table 3:** Analysis tool of the E12 genome components.

Tools	Function
GeneMarkS^36^	Predicted coding genes
Blastn database^37^	Annotated coding genes
RepeatMasker^38^	Predicted interspersed nuclear elements (INEs)
Tandem Repeats Finder^39^	Predicted tandem repeat (TR)
tRNA scan SE^40^	Predicted non-coding RNAs (ncRNAs)
RNAmmer^41^	
Rfam database^42^	
Island Path-DIOMB^43^	Predicted genomics islands (GIs)
phiSpy^44^	Predicted prophage
CRISPRdigger^45^	Predicted clustered regularly interspaced short palindromic repeat sequences (CRISPR)

### Gene function annotation

See Table 4.

**Table 4:** Analysis of the E12 genome function.

Name of database	Function
Gene Ontology (GO)^46^	A classification system for predicting gene function
Kyoto Encyclopedia of Genes and Genomes (KEGG)^47^	Analysis of gene product metabolic pathways in cells
Cluster of Orthologous Groups of proteins (COG)^48^	Phylogenetic relationship classification of encoded proteins was constructed
Non-Redundant Protein Database (NR)^49^	Annotate species information, count the number of genes
Transporter Classification Database (TCDB)^50^	Classification of membrane transporter proteins
Swiss-Prot^51^	Annotate protein structural domain functions
Carbohydrate Active Enzymes Database (CAZy)^52^	Carbohydrate-active enzymes are classified into the corresponding protein families
SignalP^53^	Predicted secreted proteins of encoded genes
TMHMM^53^	
EffectiveT3^54^	Predicted secretory system effector protein
antiSMASH^55^	Prediction of secondary metabolites
Antibiotic Resistance Genes Database (ARDB)^56^	Annotation of drug-resistant gene functions
Comprehensive Antibiotic Research Database (CARD)^57^	
Virulence Factors of Pathogenic Bacteria (VFDB)^58^	Note the function of the virulence gene
Circos^59^	Demonstration of the genome

### Ethics approval

In this study, cervical dislocation was performed as a method of euthanasia in experimental mice. The mouse was fixed on the lid of the feeding box, one hand grasps the mouse's tail and pulls it backward with a little force, while the thumb and forefinger of the other hand presses down on the head with a quick force, or uses surgical scissors or tweezers to press down quickly on the mouse's neck, and the two hands forcefully dislocates the cervical vertebrae, which results in the disconnection of the spinal cord from the medulla oblongata.

All animal experiments were performed according to the regulations of the Administration of Affairs Concerning Experimental Animals in China. The experimental protocol was approved by the Animal Welfare and Research Ethics Committee of the Inner Mongolia Agricultural University (Approval ID: NND202103).

### The ARRIVE essential 10

#### Study design 1 for each experiment, provide brief details of study design

We randomly divided the mice into three test groups (replicate groups) and one control group of four mice each (Total of 16 mice.). The mice in the test groups were given 0.4 mL of E12 at a concentration of 1.5 × 10^8^ CFU/mL, while an equal amount of saline was given to the mice in the control group. We observed the mice continuously for 3 d, recorded the time points of onset and death, and dissected the mice immediately after death to complete clinical observation and microscopic examination.

#### Sample size 2

We randomly divided the mice into three test groups (replicate groups) and one control group of four mice each (Total of 16 mice.). The mice in the test groups were given 0.4 mL of E12 at a concentration of 1.5 × 10^8^ CFU/mL, while an equal amount of saline was given to the mice in the control group. We observed the mice continuously for 3 d, recorded the time points of onset and death, and dissected the mice immediately after death to complete clinical observation and microscopic examination.

#### Inclusion and exclusion criteria 3

After administering 0.4 mL of E12 at a concentration of 1.5 × 10^8^ CFU/mL to the three groups of mice, observations were made at intervals of 4 h, mainly to see whether the mice in the test groups had any vital signs (respiration, pulse, and heartbeat). The absence of vital signs in the test group was used as the criterion for this test. The results of this test showed that the mice in the three test groups died within 9–12 h.

#### Randomisation 4

We have used randomized allocation where SPF mice that have been acclimatized to their environment are randomized into 4 groups of 4 mice each. Confounding factors were not controlled for in this experiment.

#### Blinding 5

After administering 0.4 mL of E12 at a concentration of 1.5 × 10^8^ CFU/mL to the three groups of mice, observations were made at intervals of 4 h, mainly to see whether the mice in the test groups had any vital signs (respiration, pulse, and heartbeat). The absence of vital signs in the test group was used as the criterion for this test. The results of this test showed that the mice in the three test groups died within 9–12 h.

#### Outcome measures 6

After administering 0.4 mL of E12 at a concentration of 1.5 × 10^8^ CFU/mL to the three groups of mice, observations were made at intervals of 4 h, mainly to see whether the mice in the test groups had any vital signs (respiration, pulse, and heartbeat). The absence of vital signs in the test group was used as the criterion for this test. The results of this test showed that the mice in the three test groups died within 9–12 h.

#### Statistical methods 7

Statistical software was not used in this trial.

#### Experimental animals 8

In this experiment, we used 6-week-old, physically healthy, clean-grade Kunming mice, half male and half female (16 mice in total), weighing approximately 20–22 g. The mice were purchased from the Animal Experiment Center of Inner Mongolia Medical University, Animal Certificate of Conformity No.: SCXK (Meng) 2020-0001.

The origin of the animals used in this study was from other institutions and we have obtained informed consent from the animal owners to use these animals in the study.

#### Experimental procedures 9

We randomly divided the mice into three test groups (replicate groups) and one control group of four mice each (Total of 16 mice.). The mice in the test groups were given 0.4 mL of E12 at a concentration of 1.5 × 10^8^ CFU/mL, while an equal amount of saline was given to the mice in the control group. We observed the mice continuously for 3 d, recorded the time points of onset and death, and dissected the mice immediately after death to complete clinical observation and microscopic examination.

## Results 10

Strain E12 was 100% lethal to SPF mice.

### Supplementary Information

Supplementary Information 1.

Supplementary Information 2.

Supplementary Information 3.

Supplementary Information 4.

Supplementary Information 5.

## Data Availability

The dataset generated in this study can be found in the Supplementary Material for the original data.
