# Supplementary material for: Correlation analysis of whole genome sequencing of a pathogenic Escherichia coli strain of Inner Mongolian origin
Source: Sci Rep. 2024 Jul 5;14:15494. doi: 10.1038/s41598-024-64256-5 (PMC11226720; doi:10.1038/s41598-024-64256-5)
Supplement: Supplementary file 5 — Supplementary Information 5. [file 41598_2024_64256_MOESM5_ESM.pdf]

## Results of mouse pathogenicity test of *E. coli* E12:

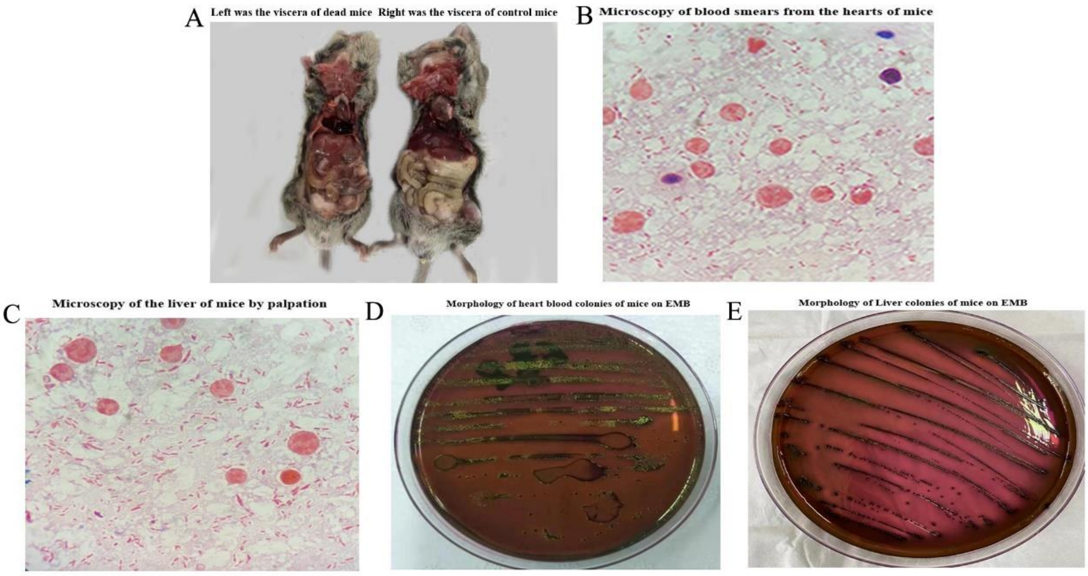


A: Left was the viscera of dead mice, right was the viscera of control mice；B: Microscopy of blood smears from the hearts of mice；C: Microscopy of the liver of mice by palpation；D: Morphology of heart blood colonies of mice on EMB；E: Morphology of Liver colonies of mice on EMB.

Fig.1 Results of mouse pathogenicity test of *E. coli* E12

## Results of gene function analysis:

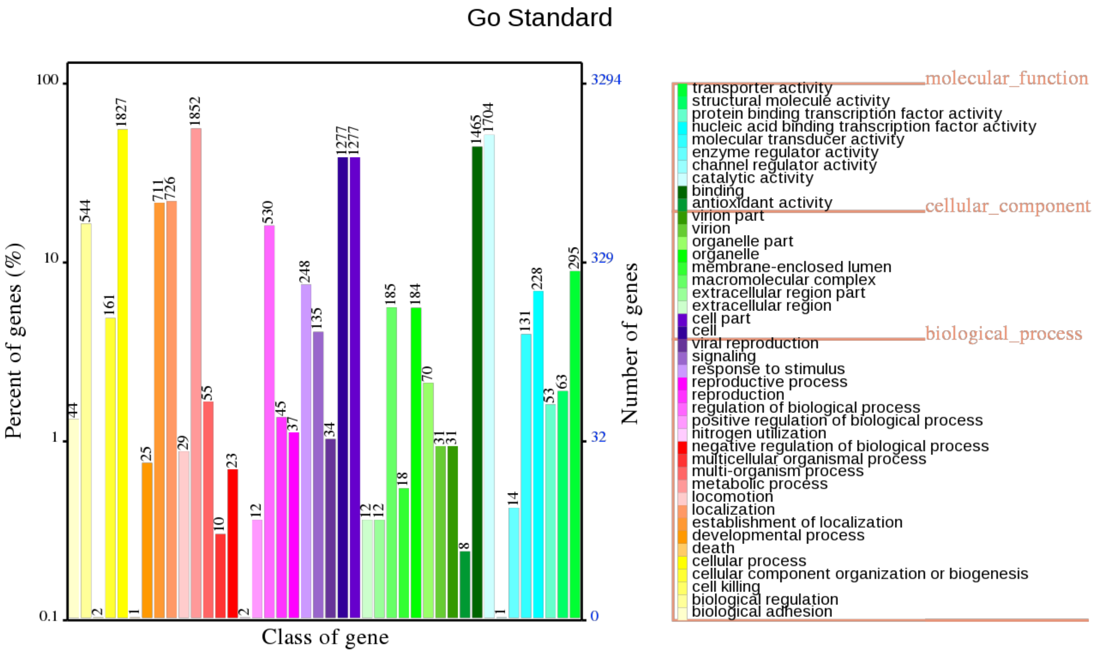


Fig.2A GO standard of E12

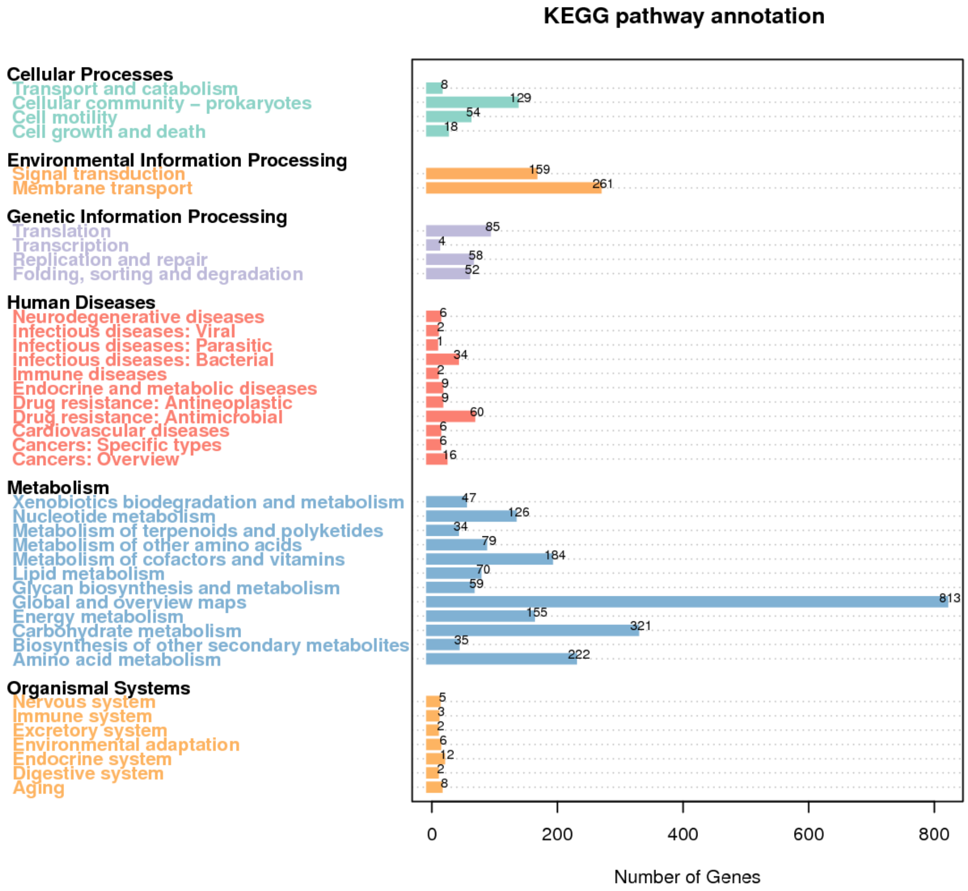


Fig.2B Kegg. functional classification of E12

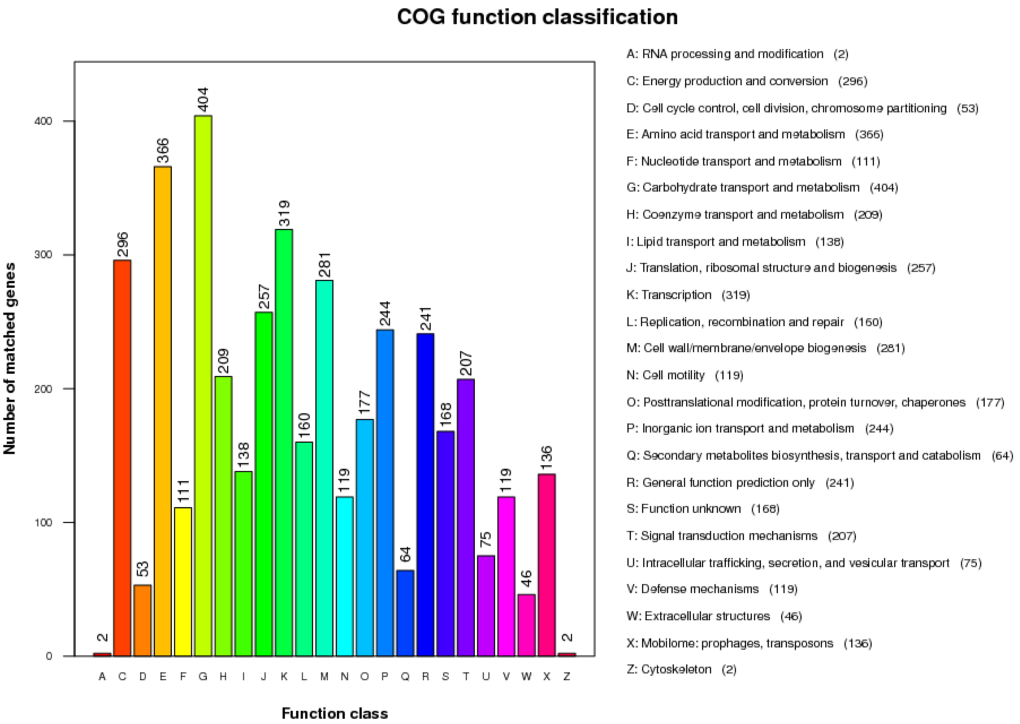


Fig.2C COG function classification of E12

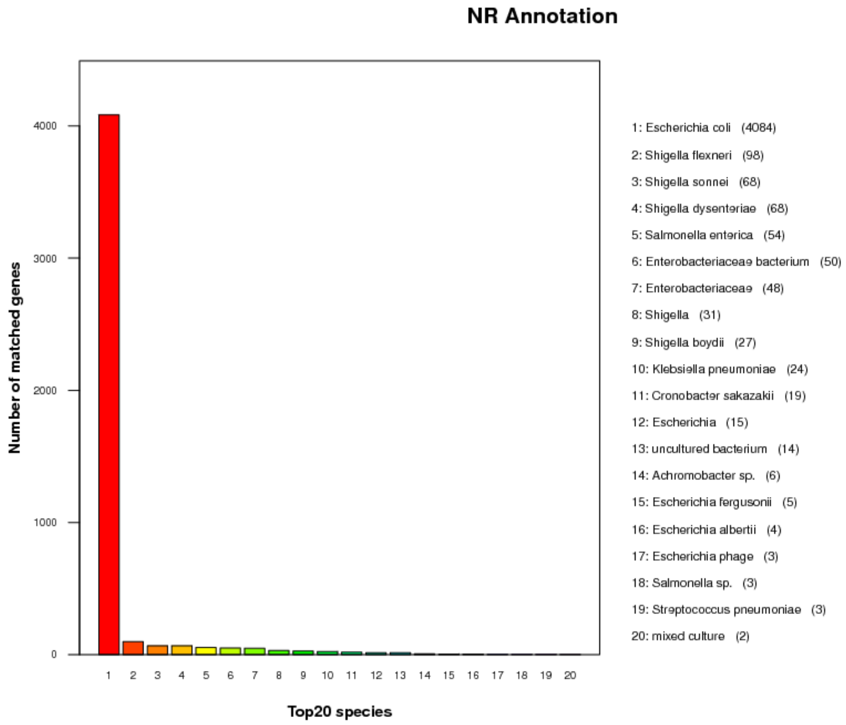


Fig.2D Nr.anno.of E12

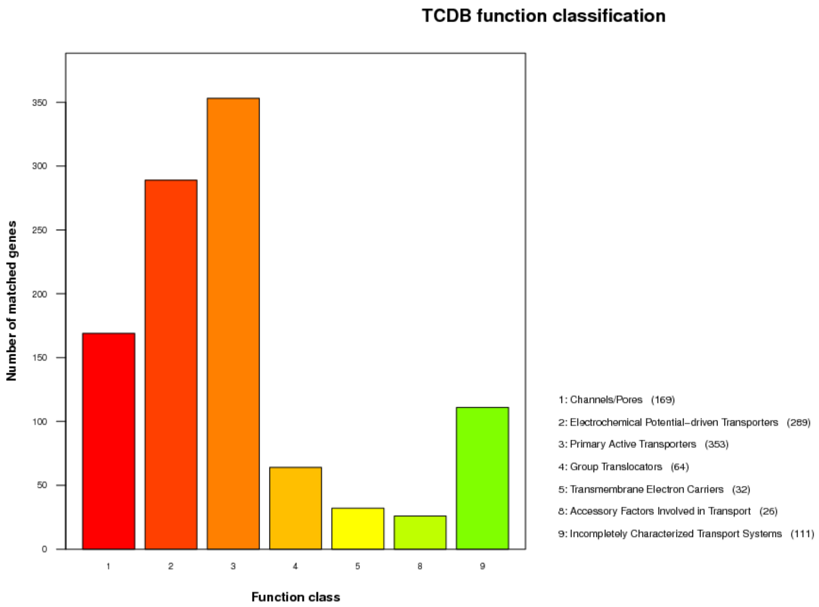


Fig.2E Tcdb.class.catalog of E12

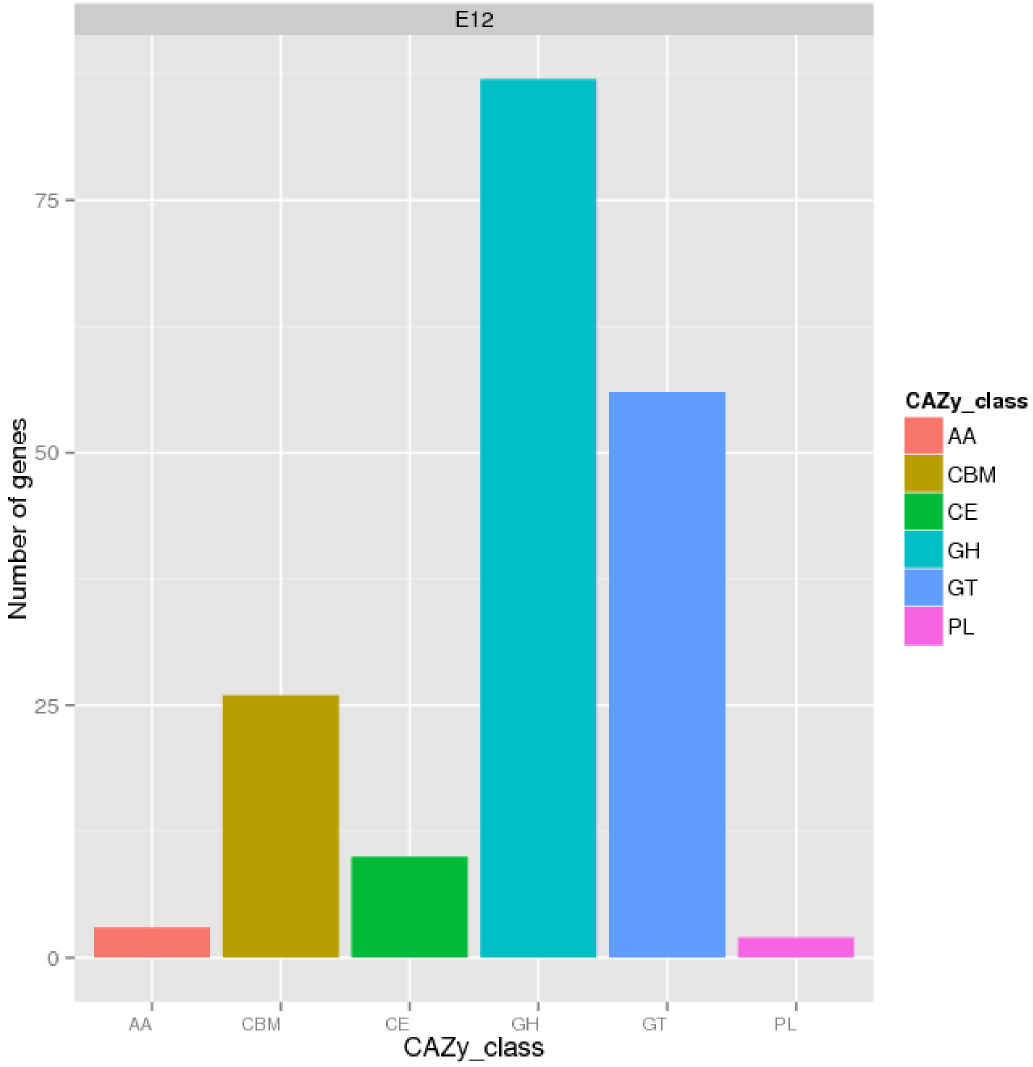


Fig.2F Single_CAZy_class of E12

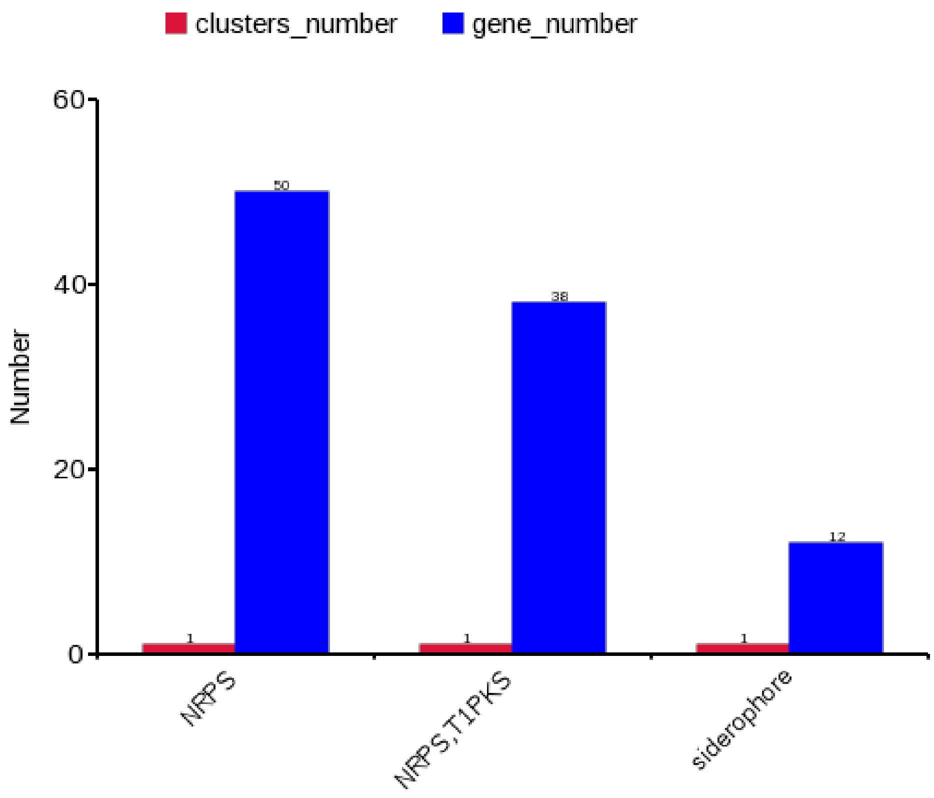


Fig.2G Cluster_num of E12

A; GO gene functional classification of E12；B: KEGG metabolic pathway classification of E12；C: COG functional classification of E12；D: NR database species annotation of E12；E: TCDB functional classification of E12；F: Classification diagram of the KEGG metabolic pathway of E12;

G: Classification diagram of the KEGG metabolic pathway of E12

Fig.2 Gene function analysis of E12
